# Chikungunya Virus Infection and Gonotrophic Cycle Shape *Aedes aegypti* Oviposition Behavior and Preferences

**DOI:** 10.3390/v15051043

**Published:** 2023-04-25

**Authors:** Margaux Mulatier, Antoine Boullis, Christelle Dollin, Gerardo Cebrián-Torrejón, Anubis Vega-Rúa

**Affiliations:** 1 Laboratory of Vector Control Research, Pasteur Institute of Guadeloupe—Lieu-dit Morne Jolivière, 97139 Les Abymes, France; a.boullis@gmail.com (A.B.);; 2 COVACHIM-M2E EA 3592 Laboratory, Université des Antilles, CEDEX, 97157 Pointe-à-Pitre, France

**Keywords:** mosquito, oviposition, behavior, gonotrophic cycle, infection, chikungunya

## Abstract

Targeting gravid females through chemical lures is a promising strategy in vector control; however, it requires the understanding of the factors susceptible to alter female oviposition behavior. Here, we evaluated the effect of infection with chikungunya virus (CHIKV) and the number of gonotrophic cycles (GCs) on oviposition activity in *A. aegypti*. Dual choice oviposition assays were performed, where dodecanoic acid, pentadecanoic acid, *n*-heneicosane and a *Sargasssum fluitans* (Børgesen) Børgesen extract were tested in uninfected females and females infected with CHIKV, at the 1st and 2nd GC. Infected females displayed a lower percentage of oviposition and a higher number of eggs laid at the 1st GC. Then, the combined effects of GC and CHIKV were observed on oviposition preferences, with a chemical-dependent effect. For instance, the deterrent effect of *n*-heneicosane and pentadecanoic acid increased at the 2nd GC in infected females. These results allow for a deeper understanding of the mechanisms involved in oviposition site selection and highlight the need for taking into account physiological stage changes to increase the control programs’ efficacy.

## 1. Introduction

*Aedes*-borne arboviral infections are responsible for a heavy burden in the intertropical regions where they circulate. In most cases, these diseases are attributed to the main vector *Aedes aegypti*. In addition, local circulation of these arboviruses is alarmingly being detected in temperate zones [1,2,3,4] due to the spread of *Aedes albopictus* to higher latitudes [5,6]. Because insecticide resistance mechanisms are dramatically spreading among vector populations [7], alternative and sustainable control tools are needed for controlling mosquito densities and maintaining transmission below an acceptable level. In the toolbox available for integrated vector management, traps against gravid females are promising because they target a cohort that has had at least one previous blood meal and is therefore most likely infected with pathogens [8,9]. In such traps, the choice of the attractant is a crucial parameter for guaranteeing both the efficacy and specificity. In this context, the study of mosquito chemical and behavioral ecology is compulsory for understanding their oviposition preferences in order to identify species-specific oviposition mediators, as well as to characterize the factors susceptible to modify these preferences. For instance, in nature, mosquitoes experience several physiological changes that are particularly relevant in the context of gravid traps such as age, experience, the number of gonotrophic cycles and infection with pathogens. Yet, although these tools are actually expected to target infectious females, their efficacy is always assessed against uninfected ones and, more generally, the impact of infection on the epidemiological outcome of control tools against vector mosquitoes remains largely unexplored.

Infection with arboviruses has been shown to alter mosquito physiology, sensory perception, and behavior. *A. aegypti* mosquitoes infected with serotype 2 of dengue virus (DENV-2) exhibit reduced motivation to feed and increased duration of blood intake [10,11,12], although other authors observed contrasted results [13]. Infection with DENV-2 can also affect mosquito fitness by reducing survival and the number of eggs laid [12], as well as by increasing the locomotor activity of *A. aegypti* [14]. Modulation of locomotor activity has also been observed with DENV-1, where infection either increased or decreased the locomotor activity depending on the incubation period [15]. Similar observations have been carried out with La-Crosse virus, where infected *A. triseriatus* and *A. albopictus* tend to probe more and engorge less than uninfected individuals [16,17]. Additionally, infection with chikungunya virus (CHIKV) was shown to reduce survival in certain *A. aegypti* [18] and *A. albopictus* populations [19]. It has also sometimes been associated with a reduced number of eggs laid [18] and egg hatching rate [20] in *A. aegypti*, as well as with a shortened time before egg laying in some *A. albopictus* populations [21]. The effect of infection with arboviruses on the oviposition behavior has only been investigated once in *A. aegypti*, where females trained to oviposit in skatole lose their preference for this compound when infected with DENV-2. This effect was not observed in females trained to oviposit in water, suggesting an influence of virus infection on learning and memory processes [22]. In this context, alteration in behavior and oviposition preferences are susceptible to occur and might be attributed to (i) alteration in the central nervous system and (ii) alteration in the perception/integration of the chemical stimuli. It is worth noting that, whereas the effect of dengue and malaria infection on host seeking and blood feeding behaviors have cornered the research interest over the last 20 years, chikungunya infection and oviposition behavior are two components that have been considerably left behind. However, chikungunya is currently a public health issue in over 60 countries around the globe [23] and is considered to be among the most problematic mosquito-borne diseases worldwide [24]. Similarly to DENV, CHIKV disseminates into the hemolymph [25], where it is able to invade the brain and sensory organs [26], suggesting a potential impact on the sensory behavior. In the context of infection and oviposition behavior, taking into account female gonotrophic cycle (GC) also deserves interest from an epidemiological point of view. Indeed, it has been shown that when females are exposed to a non-infectious blood meal a few days after the first infectious one containing CHIKV and oviposition, the proportion of mosquitoes with disseminated infection, as well as those able to transmit the virus, increased compared to mosquitoes that did not received a second blood meal [27]. This observation suggests that a second blood meal may favor the presence of the virus in secondary tissues such as those implied in chemosensory perception, therefore influencing mosquito behavior. Finally, the metabolic changes occurring in females across GC might induce changes in behavior and in oviposition preferences, which has, to the best of our knowledge, not been investigated. Consequently, with the need for implementing optimized traps against *Aedes* mosquitoes, it is compulsory that they receive validation under a scenario that takes into account the potential interplay between mosquito infection, gonotrophic cycle, oviposition behavior and preferences.

In this context, this study aims to evaluate the effect of CHIKV infection on the oviposition preferences of gravid *A. aegypti* females after the first and the second gonotrophic cycles. As *A. aegypti* females rely on olfactory and gustatory semiochemicals to select suitable oviposition sites [28,29,30], herein, the response to the following candidates previously identified as oviposition mediators was investigated: dodecanoic acid, pentadecanoic acid (stimulants, tactile cues) [31,32], *n*-heneicosane (attractant, volatile) [33,34], as well as extracts of *Sargasssum fluitans* (Børgesen) Børgesen macroalgae (repellent, volatile).

## 2. Materials and Methods

### Experimental Design

- Mosquito colony

A metapopulation of *A. aegypti* was established by sampling larvae from July to August 2019 in 5 different localities of Guadeloupe, French West Indies: Les Abymes, Pointe-à-Pitre, Deshaies, Saint-François, and Anse Bertrand [32]. Mosquito rearing took place under laboratory conditions of 26 ± 1 °C and 40–60% relative humidity (RH) with a light: dark photoperiod of 12:12 h. Larvae were reared in groups of about 200 individuals in 1 L of dechlorinated tap water and were fed with rabbit pellets. Adults were given ad libitum access to a 10% sucrose solution. Experiments were performed on the generations 8 to 11 after laboratory colonization.

- Chemicals

Compounds were selected based on the published literature showing their influence on oviposition under laboratory assays, and because their mode of perception is known. Synthetic compounds (Sigma Aldrich Inc., St. Louis, MO, USA) were *n*-heneicosane (CAS 629-94-7, purity 98%), dodecanoic acid (CAS 143-07-7, purity 99%) and pentadecanoic acid (CAS 1002-84-2, purity 99%). They were diluted in *n*-hexane (HPLC grade; Carlo Erba reagents, Milano, Italy).

- *Sargassum fluitans* (Børgesen) Børgesen extracts

Previous observations showed that extracts of the macroalgae *Sargassum fluitans* (Børgesen) Børgesen induce deterrent effects on mosquito oviposition, most probably through volatile signaling (Table S2). *Sargassum* crude material was collected along several shores of Guadeloupe from September 2020 to June 2021. *S. fluitans* was visually sorted among the different *Sargassum* species using morphological criteria, as previously described [35]. Volatile organic compounds (VOCs) of the extract (i.e., hydrolates) were separated by steam distillation using 1 kg of fresh material in 1 L of water at 100 °C for 1 h at the University Institute of Technology (University of Antilles), Saint Claude, Guadeloupe. Hydrolates were kept at −20 °C until further use.

- Viral strain

CHIKV strain was isolated in 2014 from the sera of a patient in Guadeloupe and confirmed by RT-PCR. The partial sequencing of CHIKV NS1 gene (accession number: LR792670.1) showed 97.7% identity with a strain from Suriname (accession number: KY435463.1) isolated during the CHIKV outbreak in 2014. Viral stocks were produced using a multiplicity of infection of 0.1, following two passages on African green monkey kidney Vero cells (ATCC, ref. CCL-81). The initial viral titre was estimated by cytopathic effect (CPE) examination using serial 10-fold dilutions on Vero cells and was expressed as a median tissue culture infectious dose (TCID50/mL). Viral stocks (initial titre 5.62 × 10^7^ TCID50/mL) were kept at −80 °C until use.

- Direct membrane feeding assays (DMFAs)

Batches of mated *A. aegypti* females (7–10 days old) were starved for 24 h and were then allowed to feed either with infectious or non-infectious blood (control) for 30 min. Infectious blood meal (titre 10^7^ TCID50/mL) was prepared with 1.4 mL of washed rabbit erythrocytes, 700 μL of viral suspension (diluted on Dulbecco’s Modified Eagle Medium (DMEM) containing 2% of fetal bovine serum (FBS) and adenosine triphosphate (ATP; Sigma-Aldrich, Germany) as a phagostimulant at a final concentration of 5 mM. Non-infectious blood meal was prepared with 1.4 mL of blood, 700 μL of DMEM with 2% FBS and ATP at 5 mM. 

**Figure 1:**
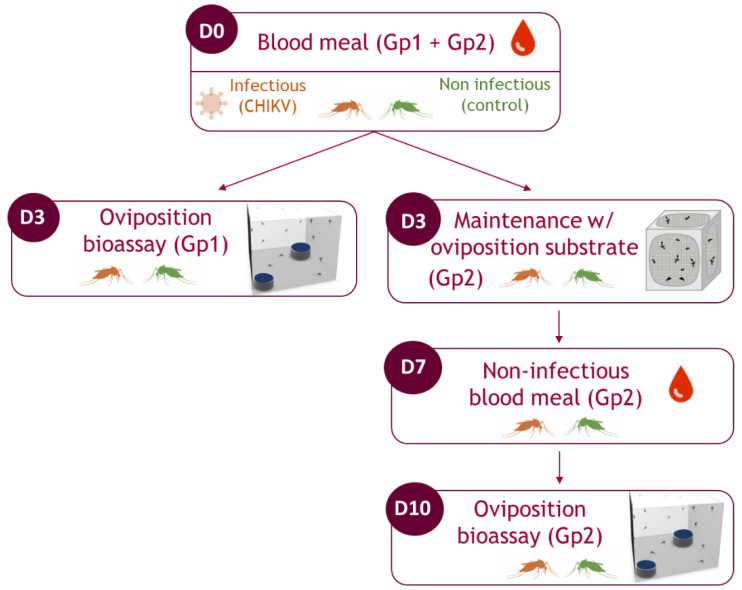
Experimental procedure used in this study. Gp1: females tested at the 1st gonotrophic cycle. Gp2: females tested at the 2nd gonotrophic cycle. D0, D3, D7 and D10 = day 0, day 3, day 7 and day 10 after the first blood meal. Red and green mosquitoes correspond to infected and non-infected females, respectively.

Fully engorged mosquitoes were sorted and maintained in cardboard containers in a climatic chamber (Memmert, Schwabach, Germany) at 28° ± 1 °C, in a 12 h:12 h light:dark cycle with 60–80% humidity, and with a 10% sucrose solution provided ad libitum. Then, each female batch (i.e., exposed to infected blood (I) and control (NI)) was split into two groups (Figure 1):

- The first group (Gp1) was tested at the first gonotrophic cycle (1st GC). Oviposition bioassays were performed three days after the first blood meal.
- The second group (Gp2) was tested at the second gonotrophic cycle (2nd GC). To conduct this, females were maintained with 10% sucrose ad libitum and were allowed to oviposit. A second, non-infectious blood meal (prepared following the same protocol described above for the control group), was provided to females 7 days after the first blood meal. Oviposition assays for this group were carried out three days after the second blood meal.

- Two-choice oviposition assays

Experiments were performed in the BSL-3 facilities of the Pasteur Institute of Guadeloupe, at 26 ± 1 °C and 60 ± 10% RH. Ceramic oviposition test bowls (Ø: 8 cm) were used for these assays. Before each trial, bowls were soaked overnight in alkaline detergent (RBS T105; chemical products R. Borghgraef, Brussels, Belgium), then abundantly rinsed and sterilized at 100 °C for 1 h. After serial dilutions of the synthetic test compounds in *n*-hexane, 100 μL of preparation was added to 100 mL of ultrapure water in the oviposition test bowl to obtain the required concentration. Test bowls were kept at room temperature for 5 min to solvent evaporation. Concentrations were chosen according to the most effective dose evidenced in previous studies: *n*-heneicosane (10 ppm) [33,36], pentadecanoic acid (10 ppm) [32], dodecanoic acid (100 ppm) [31]. Control bowls received 100 mL of ultrapure water supplemented with 100 µL of solvent (*n*-hexane). For each replicate, one control bowl and one bowl containing the tested solution were randomly placed at opposite corners of a 30 × 30 × 30 cm test cage (MegaView Science Education Services Co., Taiwan, China), with a strip of filter paper (Whatman^TM^, USA, n° 2300 916) partially immerged into each bowl to serve as an oviposition substrate. For each replicate, 10 gravid females were released into the cage. After 24 h, bowls and papers were removed from the cage and eggs were visually counted under a binocular magnifier to assess female preferences. Each chemical and condition was tested 6 times, for which the position of the bowls in the cage was randomly attributed. After the assay, females were immediately cold-anesthetized and individually kept at −80 °C.

- Confirmation of responding females and dissection

Females were examined for the presence of retained eggs by post-mortem ovary dissections. Briefly, each female was dissected under a binocular magnifier by gently severing the abdominal segments VII and VIII. Mosquitoes with >9 mature eggs were considered as gravid and were classified as non-parous [37]. For females classified as responders and exposed to CHIKV-infectious blood meal, the head was separated from the abdomen, after which they were separately ground in 300 μL of DMEM supplemented with 2% FBS for 30 s. Samples were kept at −80 °C until plaque assays.

- Assessment of sample infectious status and viral loads

The head homogenates from female responders exposed to CHIKV-infected blood were serially diluted and inoculated onto Vero cells monolayers in triplicate in 96-well plates. After incubation at 37 °C and 50% CO_2_ for 3 days, plates were stained for 30 min using a solution of crystal violet (0.2% in 10% formaldehyde and 20% ethanol) and rinsed under tap water. Evidence of the CHIKV infection was assessed by the detection of CPE and the viral titre expressed in TCID50/mL. If the heads were negative for CHIKV, the bodies were assayed in a plaque assay. The infection rate (IR) corresponds to the proportion of mosquitoes with infected bodies among those tested (responders), while the dissemination rate (DR) refers to the proportion of mosquitoes with infected heads among those having an infected body.

- Electroantennography assays

Electroantennography (EAG) assays were performed to test for the effect of *n*-heneicosane, dodecanoic acid and *S. fluitans* extracts on the antennal responses of non-infected gravid *A. aegypti* females from our mosquito colony. Females’ response to pentadecanoic acid has already recently been published [32]. EAG assays were conducted following the method previously described [32]. After stabilization of the recording signal, the presentation of a positive control (100 µg of 1-octen-3-ol) allowed for us to confirm the correct assembly of the antennae. For *n*-heneicosane and dodecanoic acid, 5 µL of the solution containing 100 µg of compound diluted in *n*-hexane was presented individually to antennae after solvent evaporation. For the negative control, 5 µL of n-hexane was presented to the antennae. For *S. fluitans*, 20 µL of pure hydrolate was used, and an air puff was presented to the antennae as the negative control. Each female antennae was exposed to only one compound (either *n*-heneicosane, dodecanoic acid or *S. fluitans* hydrolate) and the corresponding controls.

- Statistical analyses

All statistical analyses were performed using the software R 3.3.2 [38]. Females negative for infection and non-responders (i.e., that did not lay eggs) were removed from the analysis. Infection rate, dissemination rate and percentage of responding females were compared between groups by using a generalized linear mixed model (GLMM, lme4 package), with “replicate” coded as a random factor. The mean oviposition activity index (OAI) [39], with values ranging from +1 to –1, was calculated for each trial as: OAI = (NT − NC)/(NT + NC), where NT indicates the number of eggs laid in the treatment solution and NC indicates the number of eggs laid in the control solution.

For these assays, a positive OAI value indicates a preference toward the treated solution, whereas a negative OAI value indicates aversion. The mean number of eggs laid and the OAI were analyzed and compared between the infectious status and gonotrophic cycle by using a linear mixed-effects model (lmer function, lme4 package) with replicate and cage coded as random factors. The interaction between infectious status and gonotrophic cycle was always tested. Post hoc comparisons between the four groups of treatment at the second exposure were performed using multiple comparisons (Tukey’s tests, multcomp package). Model selection was performed using AIC and analysis of the residuals (RVAideMemoire package), with non-significant interactions removed from the model. EAG data were analyzed by comparing the response of each compound with those from the solvent (µV) by using a linear mixed-effects model (lmer function, lme4 package, “individual” coded as random factor). 

## 3. Results

A total of 814 females were included in this study and tested across 11 replicates, among which 612 ovipositing females were identified. 

### 3.1. Gonotrophic Cycle Impacts CHIKV Dissemination in Aedes aegypti

**Figure 2:**
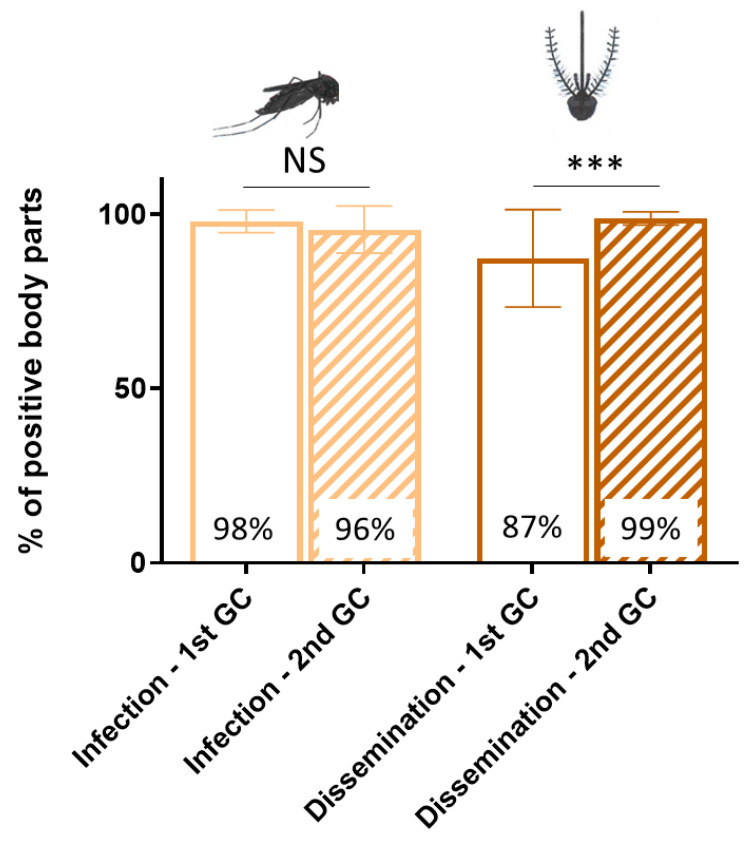
Infection and dissemination rates displayed by *Aedes aegypti* at the 1st and 2nd gonotrophic cycles following oral exposure to chikungunya virus at 10^7^ TCID50/mL. Infection rate corresponds to the proportion of mosquitoes with infected bodies among those tested, while dissemination rate refers to the proportion of mosquitoes with infected head among those having an infected body. GC = Gonotrophic cycle. Data show the mean with ±95% confidence intervals (95% CI). Asterisks indicate significant differences (chi-squared tests, NS: non-significant; *** *p* < 0.001). N = 459 females across 11 replicates.

High CHIKV infection and dissemination rates were obtained following CHIKV ingestion by *A. aegypti* mosquitoes. A mean of 97% positive bodies was estimated, with no significant difference on infection rates between the 1st GC (mean 98%; 95% CI [94.7–101.2]) and the 2nd GC (mean 96%; 95% CI [88.8–102.3]), (X^2^ = 1.18, Df = 1, *p* = 0.28). CHIKV dissemination was significantly higher at the 2nd GC (99%; 95% CI [96.9–100.7]) when compared to the 1st GC (87%; 95% CI [73.4–101.4]), (X^2^ = 9.65, Df = 1, *p* = 0.02) (Figure 2). 

### 3.2. Infection with CHIKV Impacts Egg-Laying Behavior at the First GC 

**Figure 3:**
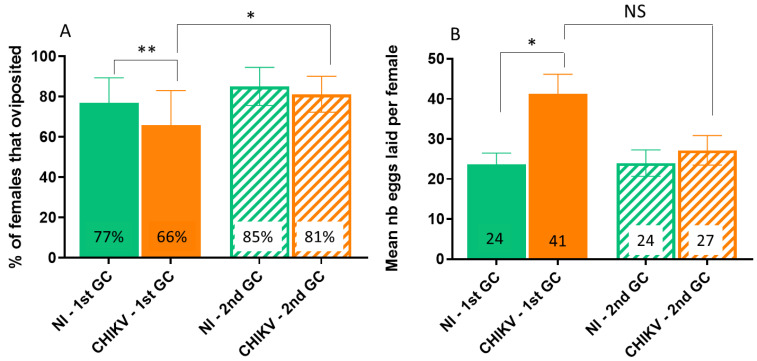
The percentage of females that oviposited (**A**) and the mean number of eggs laid per female (**B**) for non-infected (NI) and infected (CHIKV) females at the 1st and 2nd gonotrophic cycles (GC). Proportion data (**A**) show the mean with ±95% confidence intervals (95% CI), and count data (**B**) show the mean ±S.E.M. Data of the four chemicals were pooled together for this analysis. Asterisks indicate significant differences (chi-squared tests, NS: non-significant; * *p* < 0.05; ** *p* < 0.01). N = 814 females across 11 replicates.

Irrespectively of the chemical stimuli, the percentage of responding females (i.e., that oviposited) at the 1st GC was higher (mean 77%; 95% CI [64.39–89.38]) for the uninfected group (NI) than for the infected counterparts (I) (mean 66%; 95% CI [48.77–83.01]), (X^2^ = 5.97, Df = 1, *p* = 0.01). At the 2nd GC, no significant differences regarding responding females were observed between infected and uninfected groups (X^2^ = 3.3, Df = 1, *p* = 0.07), with a mean of 85% (95% CI [75.61–94.51]) for responding females for NI, and a mean of 81% (95% CI [72.32–90.15]) for the I group. For the I group, the percentage of responding females significantly increased at the 2nd GC (X^2^ = 4.14, Df = 1, *p* = 0.04). Overall, both the GC (1st GC: 71.5%; 2nd GC: 83%; X^2^ = 5.5, Df = 1, *p* = 0.02) and the infection status (NI group: 81%; I group: 73.5%; X^2^ = 9.09, Df = 1, *p* = 0.002) significantly affected the proportion of responding females (Figure 3A). 

### 3.3. Infection with CHIKV Increases Egg Batch Size

The mean number of eggs laid per responding female was influenced by significant interactions between the infectious status and the gonotrophic cycle (X^2^ = 5.58, Df = 1, *p* = 0.02), with a higher number of eggs laid by infected females than by uninfected counterparts (means of 34 and 24, respectively, when grouping both GC). At the 1st GC, significantly more eggs were laid by the I group (41 ± 4.9) than by the NI (24 ± 2.7), (X^2^ = 4.54, Df = 1, *p* = 0.03). At the 2nd GC, a trend was observed for more eggs laid in the I group (27 ± 3.7) than in the NI group (24 ± 3.3), but this was not significant (X^2^ = 0.17, Df = 1, *p* = 0.7). For the I group, the number of eggs laid decreased by a third in the 2nd GC, even though this difference was not significant (X^2^ = 0.01, Df = 1, *p* = 0.9) (Figure 3B). When considering the whole tested females (grouping responding + not responding ones), the mean number of eggs per tested female was not significantly different between the infected group and the uninfected one (X^2^ = 2.35, Df = 1, *p* = 0.1). 

### 3.4. Infection and Gonotrophic Cycle Interaction Lead to Chemical-Dependent Effects on Oviposition Preferences

#### 3.4.1. Dodecanoic Acid

**Figure 4:**
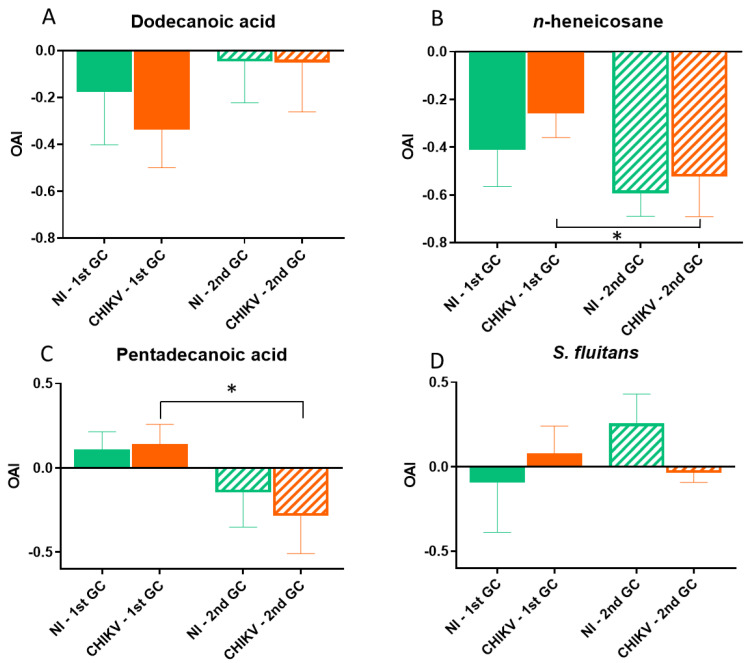
Oviposition activity index (OAI) of *A. aegypti* for non-infected (NI) and infected (CHIKV) gravid females towards dodecanoic acid (**A**), *n*-heneicosane (**B**), pentadecanoic acid (**C**) and *Sargassum fluitans* extract (**D**) at the 1st and 2nd gonotrophic cycles (GC). Data show the mean ±S.E.M. Asterisks indicate significant differences between the 1st and 2nd GC for infected females (Tukey’s tests, * *p* < 0.05). N = 612 responding females across 11 replicates.

Whatever the infectious status and GC, the OAI values elicited by this chemical were negative, and no antennal detection was observed in EAG (F = 0.387; *p* = 0.55) (Table S1), indicating deterrence. No significant differences were observed between groups when compared separately (X^2^ test: *p* > 0.05 for all pairwise comparisons). At the 1st GC, OAI values were −0.18 (±0.22) for the NI group and −0.34 (±0.16) for the I group. At the 2nd GC, OAI values were −0.046 (±0.18) for the NI group and −0.050 (±0.21) for the I group. A significant effect of the interaction between infection and GC on the OAI was observed (X^2^ = 7.51, Df = 1, *p* = 0.006). Yet, OAI values increased in the 2nd GC compared to the 1st (means of −0.26 for the 1st and −0.048 for the 2nd) and are lower in the I group than in the NI one for both GCs (means of −0.11 for NI and −0.20 for I) (Figure 4A). 

#### 3.4.2. *n*-Heneicosane

For this compound, a significant effect of the GC was observed on egg-laying behavior, where the percentage of responsive females was significantly lower at the 2nd GC for both infectious groups (X^2^ = 14.82, Df = 7, *p* = 0.04). 

OAI mean values were −0.41 (±0.15) for the NI group and −0.26 (±0.10) for the I group at the 1st GC, and −0.59 (±0.10) and −0.52 (±0.17), respectively, at the 2nd GC, indicating oviposition deterrence for this compound. There was a significant effect of the GC on the OAI, with values being significantly lower at the 2nd than at the 1st GC for both groups (means of −0.34 at the 1st and −0.56 at the 2nd) (X^2^ = 7.86, Df = 1, *p* = 0.005). Pairwise comparisons also showed a stronger deterrent effect at the 2nd GC than at the 1st GC for the I group (X^2^ = 5.6, Df = 1, *p* = 0.02) (Figure 4B). No antennal detection was recorded in EAG for this compound (F = 0.002; *p* = 0.96) (Table S1).

#### 3.4.3. Pentadecanoic Acid

At the 1st GC, OAI values were positive for both groups, indicating oviposition stimulation (0.11 for NI (±0.11) and 0.14 (±0.12) for I). This effect was reversed at the 2nd GC for both groups—the compound became deterrent with negative OAI values observed (−0.15 (±0.20) for NI and −0.28 (±0.23) for I). For this compound, there was a significant increase in the deterrent effect between the first and the second GC (0.13 for the 1st and −0.22 for the 2nd) (X^2^ = 4.18, Df = 1, *p* = 0.04) independently of the infectious status, even if this trend was more marked for the I group (X^2^ = 4.74, Df = 1, *p* = 0.03) (Figure 4C). As stated in Boullis et al. [32], this compound elicited no antennal detection in EAG.

#### 3.4.4. *Sargassum fluitans*

The *S. fluitans* extract showed a weak effect for both groups at the 1st GC, with slight aversion for the NI group (−0.09 (±0.29)) and stimulation for the I group (0.08 (±0.16)). At the 2nd GC, the extract showed stimulation for the NI group (0.26 (±0.17)) and a weak effect for the I group (−0.03 (±0.060)). Although differences were noted between infectious status, OAI values were not significantly affected by infection (X^2^ = 0.27, Df = 1, *p* = 0.6) nor GC (X^2^ = 0.08, Df = 1, *p* = 0.7) (Figure 4D). EAG signals were statistically different between the females’ responses to *S. fluitans* isolates and corresponding controls (F = 73.499; *p* < 0.0001) (Table S1).

## 4. Discussion

Our results showed a high competence of the *A. aegypti* metapopulation used for CHIKV, with an infection rate exceeding 95% and dissemination of over 85% at the first GC, as soon as 3 days post-infection. These observations are in agreement with the high dissemination levels found for CHIKV in *A. aegypti* populations from the French West Indies [40], as well as with the rapid dissemination reported for this virus in *Aedes* spp. mosquitoes (from 2–3 days after the infectious blood meal) [41]. Additionally, we found that the gonotrophic cycle impacted dissemination, with significantly more CHIKV dissemination detected at the 2nd GC, thus corroborating that successive blood meals favor arbovirus dissemination beyond the midgut within mosquitoes [27]. Further studies evaluating the dissemination of a cohort, of which has not been provided with a second blood meal, would allow for us to discriminate the effects of GC and aging on virus dissemination.

Our behavioral assays allowed for the evidence-combined effects of both mosquito infection and different gonotrophic cycles on mosquito physiology as well as on oviposition preferences. First, infection with CHIKV reduced the egg-laying activity of the tested cohorts, as a lower percentage of infected females oviposited when compared to uninfected counterparts, especially at the 1st GC. To the best of our knowledge, this is the first report of a reduced percentage of oviposition in females infected with CHIKV. On the other hand, the number of eggs laid by responding females was higher in the infected group than in the uninfected one, with a stronger effect at the 1st GC. Previous observations showed either no effect of CHIKV infection on the number of eggs laid [19,20] or a lower number of eggs laid in infected females at the 1st GC but not at the 2nd [18], which contrast with our results. Possible explanations for these discrepancies could lie in the experimental design: in our study, groups of 10 females were given 24 h to oviposit and the mean number of eggs laid per female was obtained by dividing the total number of eggs by the number of responding females (that did not have any retained eggs in the ovaries). Previous studies either tested females individually [19,20] or in large groups [18], and allowed for them to oviposit for 90 min [20] or without a time limit [18,19]. For the study testing females in groups, ovary dissection was not performed; therefore, the total number of tested females was used to calculate the mean number of eggs laid per female, which can lead to an overestimation of the responding females. Design considerations have been proposed for increasing the reproducibility of oviposition experiments, which could allow for an improvement in the comparison between studies [42,43].

Taken together, these observations suggest that infection with CHIKV might induce a fitness cost on mosquito oviposition pathways, as seen elsewhere [18]. Reproduction and immunity can be mutually constraining, as immune activation has been shown to be associated with a reduced reproductive capacity in many insects (for the review, see [44]). Under this scenario, infection with CHIKV would be expected to reduce the number of ovipositing females and/or the number of eggs laid. Such effects may also vary according to environmental conditions and to different mosquito and virus genotype combinations. The oviposition success reduction observed here as early as in the 1st GC may suggest a higher fitness cost of CHIKV infection on *A. aegypti* mosquitoes when compared to that of other arboviruses such as DENV, for which a negative impact on *A. aegypti* oviposition was only detected by the 3rd and 4th GCs, and combined with an age-dependent effect [12].

It is noteworthy that other models predict that an organism would increase its reproductive effort when the survival threat is inherent to infection [45], even in the case of non-pathogenic immune stimulation [46]. In our experimental set-up, the reduced proportion of responding females in the infected group is counterbalanced by the higher number of eggs laid by this group. Consequently, these physiological modifications do not translate into a change in entomological indicators, as the mean number of eggs per female when considering the whole tested cohort (responders + not responders) remain the same. Therefore, further studies need to be performed in order to understand the physiological processes and trade-offs that take place between CHIKV infection and the egg-laying pathway. For instance, testing the viability of eggs would allow for a deeper understanding of the mechanisms involved [20,45]. Our data also revealed that, for both infectious groups, a higher percentage of females oviposited at the 2nd GC than at the 1st. This behavior has been frequently observed in *Aedes* mosquitoes, as females often need to take multiple blood meals before developing a batch of eggs [47,48,49]. It is worth noting that, in our study, the number of eggs laid per female was relatively low (around 25–40). Several factors could explain these observations. First, the confined conditions in the BSL-3 facility associated with a negative air pressure could have induced a stress on females. Then, several studies have shown that the blood meal source (here rabbit blood) and the addition of anticoagulant can have an impact on the fecundity [50,51,52,53]. These factors could also explain the variability between replicates observed in the percentage of females that oviposited.

The combined effects of infection and the number of gonotrophic cycles were also observed on oviposition preferences, with a chemical-dependent effect. First, an interactive effect of GC and infection was observed on the efficacy of dodecanoic acid, where the deterrent effect observed in the 1st GC (especially for the infected group) almost disappears at the 2nd GC for both infectious groups. Then, *n*-heneicosane and pentadecanoic acid showed an increase in the deterrent effect at the 2nd GC for both groups, this change being more marked for the infectious group. *S. fluitans* is the only candidate for which the valence did not significantly change either across infection nor across GC. One explanation for this could be that by being a hydrolate from algae origin, *S. fluitans* is less concentrated in semiochemicals than the other pure compounds (alkane and fatty acids) tested in this study, and might therefore elicit a lower behavioral response. Interestingly, dodecanoic acid and *n*-heneicosane appeared to be a deterrent in our study, whereas they were previously documented as a stimulant [31,34,54]. The testing of *n*-heneicosane under semi-field experiments using the same mosquito population also confirmed the deterrent effect in our mosquito population (data not shown). It is worth noting that, in the study performed by Baak-Baak and colleagues [34], the observed attractant effect was relatively low (0.09 at 10 ppm), and a deterrent effect was observed at high doses (−0.14 at 100 ppm). In the present study, lower concentrations were sufficient to induce a stronger deterrent effect. Furthermore, in this experimental set-up, *n*-heneicosane did not induce any antennal response in EAG experiments, which contrasts with previous studies showing *A. aegypti* responses in both in EAG [33,36] and olfactometers [33].

Taken together, these data allow for us to provide evidence on four parameters that are likely to modulate the behavioral output of a compound on gravid females: (i) the experimental set-up, (ii) the mosquito population used, (iii) the infectious status and (iiii) the number of GCs. Yet, when using the same doses and methodology, some compounds previously shown as a stimulant were found to be a deterrent in our conditions. Further studies need to be performed under different scenarios to understand the parameters potentially involved in these changes. For instance, in the previous study of Baak-Baak and colleagues [34], *n*-heneicosane was prepared in dichloromethane, whereas *n*-hexane was used in our study. The choice of the solvent might affect both the chemistry of the product and the behavioral effect on the tested insect. Then, the types of blood used and environmental conditions are thought to affect mosquito behavior [50,51,52,55]. Additionally, mosquito-related parameters such as genotype and microbiome are likely to explain these differences. To the best of our knowledge, there are no studies comparing the effect of an attractant or deterrent over several mosquito genotypes. Testing the effect of these parameters and using other electrophysiology techniques such as single sensillum recording would allow for us to depict which factors are likely to drive mosquito preferences. Then, CHIKV infection, together with the number of GCs, modulated the deterrence of a compound compared to uninfected females. Such changes in oviposition preferences have already been documented with dengue infection [22]. Dengue viruses have been shown to change the expression levels of host-seeking genes [56] as well as the overall neural responsiveness of *A. aegypti* antennae [15]. Changes in the neuronal network have also been recorded in the case of Zika virus infection [57]. The three compounds for which an effect of infection have been observed, dodecanoic acid, *n*-heneicosane and pentadecanoic acid, did not induce any EAG response in our setting and might be considered here as tactile cues. As CHIKV also replicates in the head [25,26] and possibly in the central nervous system, this could interfere with the behavior and signal processing of these cues. Finally, the effect of a compound strongly varies between the 1st and 2nd GC, with either an increase (*n*-heneicosane, pentadecanoic acid) or decrease (dodecanoic acid) in the deterrent effect. Similarly to infection, these differences might be associated with both changes in the perception and in the integration of the chemical stimuli, as many physiological changes occur across female GCs. For instance, *A. aegypti* females can be trained to display oviposition preferences, but these induced preferences fade after a second blood meal [22]. Because of the importance of the operational implications of such behavioral changes, further studies are crucial to understand the associated mechanisms and to determine if the preferences stabilize after the 2nd GC.

## 5. Conclusions

In the present study, we provide evidence, for the first time, on changes in the preferences of some compounds previously identified as oviposition mediators according to infection and GC. These observations could have strong implications in the area of mosquito control. Yet, the valence of oviposition disruptors used in traps against gravid females might not be linear across a mosquito’s life, suggesting that the efficacy of some compounds observed under laboratory experiments might be difficult to extrapolate under natural conditions. Additionally, traps designed to target epidemiologically relevant females (i.e., blood-fed once and infected) might be less specific as expected. Altogether, these data would explain the discrepancies observed between predictions and observations in the field. This highlights the need for developing research protocols that take into account mosquito physiology and for testing the efficacy of a candidate under different scenarios before implementing a tool in the field.

## Data Availability

All data are available on https://datadryad.org/stash/share/EF062nZ9lJNecT9s1Gm5kMogL9LDcP-BFUc1VWDxq5A, accessed on 2 March 2023.

## Supplementary Materials

The following supporting information can be downloaded at: https://www.mdpi.com/article/10.3390/v15051043/s1, Table S1: Electroantennography response (µV; ±S.E.) of gravid *Aedes aegypti* females to *n*-heneicosane, dodecanoic acid, *Sargassum fluitans* hydrolate and their respective negative controls. *p*-values presented in the table correspond to comparison of antennal responses between the negative control and the tested compound (Tukey’s post hoc tests). Table S2: Oviposition responses of *A. Aegypti* gravid females towards *S. fluitans* extract dosed at 10^−1^. OAI values ranged between −1 and 1; negative values indicate deterrence and positive values indicate stimulation. N = 125 females tested by groups of 25 females across 5 replicates.
