# Supplementary material for: Chikungunya Virus Infection and Gonotrophic Cycle Shape Aedes aegypti Oviposition Behavior and Preferences
Source: Viruses. 2023 Apr 25;15(5):1043. doi: 10.3390/v15051043 (PMC10224293; doi:10.3390/v15051043)
Supplement: Supplementary file 1 [file viruses-15-01043-s001.zip › viruses-2301658-supplementary.pdf]

**Table S1. Electroantennography response (µV; ±S.E.) of gravid *Aedes aegypti* females to *n*-heneicosane, dodecanoic acid, *Sargassum fluitans* hydrolate, and their respective negative controls.** P-values presented in the table correspond to comparison of antennal responses between negative control and the tested compound (Tukey-s post hoc tests).

| | Compound | Negative control | Statistic (F) | P.value |
|---|---|---|---|---|
| ***n*-heneicosane (n=8)** | 204 ± 22.85 | 208 ± 22.56 | 0.387 | 0.553 |
| **dodecanoic acid (n=7)** | 171 ± 22.72 | 170 ± 21.13 | 0.002 | 0.959 |
| ***S. fluitans* hydrolate (n=11)** | 273 ± 33.64 | 183 ± 26.79 | 73.499 | $6.391 \times 10^{-6}$ |

**Table S2. Oviposition responses of *Ae. Aegypti* gravid females towards *S. fluitans* extract dosed at $10^{-1}$.** OAI values range between −1 and 1, were negative values indicate deterrence and positive values indicate stimulation. N = 125 females tested by groups of 25 females across 5 replicates.

| Replicate | Nb of eggs in the treated solution | Nb of eggs in the control solution | Total nb eggs | OAI |
|---|---|---|---|---|
| 1 | 418 | 462 | 880 | −0.05 |
| 2 | 616 | 945 | 1561 | −0.21 |
| 3 | 337 | 489 | 826 | −0.18 |
| 4 | 185 | 587 | 772 | −0.52 |
| 5 | 296 | 1088 | 1384 | −0.57 |
